# Antibio-résistance des souches de *Salmonella spp* isolées d'hémocultures à Bukavu en RD Congo

**DOI:** 10.11604/pamj.2018.29.42.13456

**Published:** 2018-01-17

**Authors:** Théophile Mitima Kashosi, Archippe Birindwa Muhandule, David Lupande Mwenebitu, Nicolas Mihuhi, John Kivukuto Mutendela, Kanigula Mubagwa

**Affiliations:** 1 Laboratoire de Recherche Biomédicale et de Santé Publique, Département des sciences Biomédicales, Faculté de Médecine et Santé Communautaire, Université Evangélique en Afrique, Bukavu, RD Congo; 2 International Center for Advanced Research and Training, Bukavu, RD Congo; 3 Section Techniques de Laboratoire, Institut Supérieur des Techniques Médicales (ISTM), Bukavu, DR Congo; 4 Département de Pédiatrie, Hôpital de Panzi, Faculté de Médecine et Santé Communautaire, Université Evangélique en Afrique, Bukavu, RD Congo; 5 Service de Microbiologie, Hôpital Provincial Général de Référence de Bukavu/Université Catholique de Bukavu (UCB), RD Congo; 6 Service de Microbiologie, Cliniques Universitaires de Kinshasa, Université de Kinshasa, RD Congo; 7 Médecins d’Afrique, Coordination-Europe, Savigny Sur Orge, France; 8 Department of Cardiovascular Sciences, University of Leuven, Leuven, Belgium

**Keywords:** Salmonella, antibiotic resistance, Bukavu, Democratic Republic of the Congo, Salmonella, antibio-résistance, Bukavu, RD Congo

## Abstract

**Introduction:**

La fièvre typhoïde est un problème majeur de santé publique dans les pays en voie de développement jusqu'à ce jour à cause de la vétusté des infrastructures sanitaires et des circuits de distribution de l'eau presque inexistants. En RDC en général et à Bukavu en particulier, l'hémoculture est inaccessible à la majorité des patients. L'objectif de cette étude était d'évaluer la sensibilité de *Salmonella spp* aux antibiotiques couramment utilisés dans la prise en charge de la fièvre typhoïde à Bukavu.

**Méthodes:**

Une étude transversale étalée sur 6 mois a était organisée. Tout malade suspect de la fièvre typhoïde a été sélectionné dans l'étude. L'hémoculture était faite systématiquement chez tout malade sélectionné. L'identification de la souche bactérienne et l'antibiogramme ont été réalisés par les méthodes conventionnelles. Les antibiotiques suivants ont été testés: amikacine, amoxicilline, augmentin, aeftazidime, ceftriaxone, cefuroxime, chloramphénicol, ciprofloxacine, cotrimoxazole, doxycycline, gentamicine, négram, norfloxacine.

**Résultats:**

460 malades ont été sélectionnés dans l'étude. 144 (31,30%) hémocultures positives ont été observées. *Salmonella spp* était le germe le plus isolé (41,66%). Les souches de *Salmonella spp* isolées à Bukavu sont sensibles à la ciprofloxacine (91,7%), au ceftazidime (81,7%), ceftriaxone (80%), norfloxacine (80%), amikacine (76,6%) et au cefuroxime (73,3%). Elles restent résistantes aux autres molécules d'antibiotiques.

**Conclusion:**

Ces résultats montrent une sensibilité diminuée à plupart des antibiotiques testés. Un test d'antibiogramme est requis en cas de fièvre typhoïde pour une meilleure prise en charge.

## Abstract (en)

**Introduction:**

Typhoid fever is a major public health problem in developing countries because of the obsolescence of health infrastructure and of an almost non-existent water distribution network. In the Democratic Republic of the Congo, in general, and in Bukavu, in particular, blood culture is inaccessible to the majority of patients. This study aimed to assess the sensitivity of Salmonella spp to antibiotics commonly used for the tratment of typhoid fever in Bukavu.

**Methods:**

We conducted a cross-sectional study over a period of 6 months. Any subject suspected of having typhoid fever was included in the study. Blood culture was systematically performed in any selected patient. The identification of bacterial strain and antibiotic susceptibility tests were performed using conventional methods. The following antibiotics were tested: amikacin, Aamoxicillin, augmentin, ceftazidime, ceftriaxone, cefuroxime, chloramphenicol, ciprofloxacin, cotrimoxazole, doxycycline, gentamicin, negram, norfloxacin.

**Results:**

Our study included 460 sick subjects; blood cultures were positive in 144 (31.30%) patients. Salmonella spp was the most isolated germ (41.66%). In Bukavu, isolated Salmonella spp strains were sensitive to ciprofloxacin (91.7%), ceftazidime (81.7%), ceftriaxone (80%), norfloxacin (80%), amikacin (76.6%) and cefuroxime (73.3%). They remained resistant to other antibiotic molecules.

**Conclusion:**

These results show a decreased sensitivity to most of the antibiotics. Antibiotic susceptibility test is necessary in patients with typhoid fever for improved patient management.

## Introduction

La fièvre typhoïde (FT) reste encore aujourd'hui un problème majeur de santé publique dans les pays en voie de développement [1] contrairement aux pays industrialisés où la modernisation des infrastructures sanitaires et l'amélioration des circuits de distribution de l'eau [1,2], ont permis de la reléguer au rang des maladies rares ou importées. Ainsi, dans les pays en développement on note 150 à 1000 cas par an pour 100 000 habitants [3,4]. En 2002 par exemple, elle a été la cause d'hospitalisation de 408837 cas en Afrique [3]. A Bukavu, la morbi-mortalité liée à cette maladie est importante. En 2011 par exemple, selon l'Inspection Provinciale de la Santé (IPS/Sud Kivu) on a enregistré 19022 cas de FT, en 2012 on a observé une légère augmentation des cas jusqu'à 20325 cas alors qu'en 2013, une explosion de cas de la FT a été observée [5]. Un total de 28200 cas ont été noté en 2013, dont 2% étaient des enfants de 0-11mois, 17,5% des cas avec un âge variant entre 12 et 59mois, 27% des cas avec un âge de 6-14ans, et 53,4% des cas avec un 'âge de 15 ans ou plus [5]. Dans les pays développés, le diagnostic repose sur l'isolement du germe [3]. Mais dans les pays à faibles revenus, où les moyens de culture font défaut [5,6], le sérodiagnostic de Widal-Félix est plus effectué étant le plus économique des moyens diagnostiques. D'où l'intérêt d'une étude pouvant évaluer la sensibilité de *Salmonella spp* aux antibiotiques utilisés empiriquement dans le traitement de la fièvre typhoïde à Bukavu. L'objectif était d'évaluer la sensibilité de *Salmonella spp* aux antibiotiques couramment utilisés dans la prise en charge de la FT en province du Sud-Kivu en général et dans la ville de Bukavu en particulier.

## Méthodes

**Type et sites d'étude:** Il s'agit d'une étude transversale, qui s'est déroulée à Bukavu, coordonnée à partir du Laboratoire de Recherche Biomédicale et de Santé Publique (LRSP) de la Faculté de Médecine de l'Université Evangélique en Afrique (UEA) de juin à décembre 2014 et qui a i,pmiqué 5 formations de santé (FOSA) dont: l'Hôpital Général de Référence (HGR) de Panzi, le Centre Hospitalier de la 8^e^ Communauté des Eglises Pentecôtistes en Afrique Centrale de Cahi (CH 8^e^ CEPAC Cahi), l'Hôpital Général de Kadutu (HG Kadutu), le Centre Hospitalier de la Communauté Baptiste au Centre de l'Afrique de Nyamugo (CH CBCA-Nyamugo) et le Centre Hospitalier de la 5^e^ Communauté des Eglises Libres de la Pentecôte en Afrique (5^e^ CELPA) d'Ibanda. La ville de Bukavu est le chef lieux de la province du Sud-Kivu et compte actuellement trois communes (Ibanda, Kadutu et Bagira) avec un total de 32 quartiers repartis dans différentes communes.

**Population d'étude:** Eté inclus dans la présente étude tous les malades qui présentaient au moins 3 symptômes ou signes cliniques faisant penser à la FT et ayant consulté dans l'une de 5 formations sanitaires (FOSA). Les symptômes et signes cliniques pris en compte étaient: fièvre, douleur abdominale, céphalées, diarrhée ou constipation, asthénie et/ou typhos, anorexie, vomissements, courbatures, épistaxis, anémie, vertiges. Etait exclu de cette étude tout malade qui avait pris un ou plusieurs antibiotiques avant le prélèvement.

**Recueil des données:** Pour tout malade, les données étaient récoltées dans ces différentes FOSA par une équipe (médecin et technicien de Laboratoire). Le médecin consultant complétait la fiche de collecte des données (données anthropométriques et signes cliniques) et demandait systématiquement le Widal et les hémocultures pour tout cas inclus. Le malade était alors envoyé au laboratoire de la FOSA où étaient prélevés les différents échantillons par le Technicien. Les hémocultures étaient directement prélevés dans des flacons d'hémoculture BactAlert de Bio Mérieux; Les échantillons sanguins prélevés pour le Test de Widal étaient quant à eux traités dans chaque FOSA, en utilisant le kit TYDAL Widal Antigen set / Tulip diagnostics (P) Ltd en suivant les instructions du fabricant, et après une séance d'harmonisation des manipulations avec tous les techniciens de laboratoire de ces différentes FOSA. Les hémocultures prélevées étaient acheminées chaque jour entre 12 et 14 heures à température ambiante au Laboratoire (LRSP) par le médecin stagiaire enquêteur.

**Analyses de laboratoire:** Au LRSP, les hémocultures étaient mises en culture directement et incubées à 37°C. Seules les hémocultures positives (flacon trouble, hémolysé ou coagulé) étaient par la suite isolées sur milieux solides (Mac Conkey, milieu de Chapman et gélose au sang 5% à base de trypticase de soja) avant d'être identifiés selon le cas sur milieux d'identification en utilisant les techniques conventionnelles. Ont été retenues comme souches de *Salmonella spp*. Les souches qui avaient les caractères biochimiques suivants: glucose +, H2S +, lactose -, urée -, O-nitrophenyl-beta-D-galactopyranoside (ONPG)- et lysine decarboxylase (LDC) +[7]. Le test d'antibiogramme était effectué selon la méthode modifiée de Kirby Bauer, recommandée par l'OMS et basée sur la diffusion à partir de disques imprégnés d'antibiotiques sur gélose de Muller-Hinton [8]. Les antibiotiques suivants ont été testés: amikacine, amoxicilline, augmentin, ceftazidime, ceftriaxone, cefuroxime, chloramphénicol, ciprofloxacine, cotrimoxazole, doxycycline, gentamicine, Négram, norfloxacine. Les disques utilisés étaient de marque Cypress.

**Analyses statistiques:** Les données ainsi récoltées ont été saisies sur Excel 2010, puis analysées par Epi Info version 3.5.3. et par MedCalc Statistical software. Nous avons considéré une association entre une variable et le phénomène étudié en cas de rapport de cote (OR) supérieur à 1 avec un intervalle de confiance (IC à 95%) excluant 1 à sa borne inférieure et la valeur P inférieur à 0,05.

## Résultats

Nous avons colligé 460 échantillons. L'âge moyen (±écart type) des enquêtés était de 28,09±17,7 ans avec des extrêmes de 3 mois et 80 ans. Les hommes étaient prédominants avec un sexe ratio de 1,3. Le Centre Hospitalier 8^ème^ CEPAC Cahi a fourni les gros de l'échantillon. Les mariés et les cas avec un niveau d'étude secondaire étaient majoritaires dans l'étude. L'analyse statistique a montré une association de la fièvre typhoïde avec le jeune âge (inférieur à 15 ans), le sexe féminin ainsi que les cas sans niveau d'étude (Tableau 1). La fièvre, les douleurs abdominales et des céphalées étaient les éléments cliniques qui dominaient chez nos enquêtés. La fièvre typhoïde était associée à la fièvre, aux vomissements et aux douleurs abdominales (Tableau 2). Parlant des souches bactériennes isolées des hémocultures, les entérobactéries étaient plus dominantes avec *Salmonella ssp* en tête (Tableau 3). Les salmonelles isolées étaient sensibles à la ciprofloxacine, au ceftazidime, à la ceftriaxone, à la norfloxacine, à l’amikacine et au cefuroxime (Tableau 4).

**Tableau 1:** Caractéristiques sociodémographiques de nos enquêtés

		Fièvre typhoïde		Analyse bi variée		
Caractéristiques (n=460)	Fréq,	Pos (%)	Nég (%)	OR	IC95%	p
**Age (28,09 ans±17,72)**						
0-15 ans	70	71,4	28,6	6,40	3,54-11,56	< 0,0001
16-30 ans	235	28	72	1		
31 ans et plus	155	18	82	0,56	0,33-0,95	0,0235
Sexe						
Féminin	203	41	59	2,22	1,488-3,31	0,00008
Masculin	257	24	76	1		
**Adresse (Centre de prélèvement)**						
CH 8e CEPAC Cahi	194	22,7	77,3	0,40	0,22-0,73	0,00024
CH CBCA Nyamugo	140	27,9	72,1	0,52	0,28-0,98	0,042
CH 5e CELPA Ibanda	64	42,2	57,8	1		
HG Kadutu	62	54,8	45,2	1,66	0,82-3,36	0,155
**Niveau d’étude**						
Sans	44	54,5	45,5	3,31	1,47-7,47	0,00323
Primaire	130	36,2	63,8	1,56	0,81-3,07	0,181
Secondaire	222	25,2	72,8	0,93	0,49-1,75	0,82
Universitaire	64	26,6	73,4	1		

**Tableau 2:** Manifestations cliniques et fièvre typhoïde confirmée par hémoculture

Manifestation	n=460	Fièvre typhoïde		Analyse bi variée		
**Fièvre**	Fréquence	Pos (%)	Nég (%)	OR	IC 95%	P
Avec	325	36,9	63,1	2,71	1,61-4,59	<0,001
Sans	135	17,8	82,2			
**Diarrhée**						
Avec	96	14,6	85,4	0,45	0,23-0,86	0,013
Sans	364	27,5	75,5			
**Constipation**						
Avec	48	20,8	79,2	0,78	0,35-1,69	0,622
Sans	412	25,2	74,8			
**Asthénie**						
Avec	127	12,6	87,4	0,35	0,19 - 0,63	0,0003
Sans	333	29,4	70,6			
**Anorexie**						
Avec	109	23,8	76,2	0,94	0,55-1,59	0,896
Sans	351	25,1	74,9			
**Vomissement**						
Avec	77	40,3	59,7	2,44	1,41-4,21	<0,001
Sans	383	21,7	78,3			
**Douleurs abdominales**						
Avec	347	27,7	72,3	2,02	1,12-3,66	0,017
Sans	113	15,9	84,1			
**Céphalée**						
Avec	351	25,3	74,7	1,14	0,67-1,96	0,7
Sans	109	23	77			

**Tableau 3:** Germes isolés des hémocultures

**Hémoculture (n=144)**	Fréquence	%
Salmonella spp	60	41,7
Staphylocoque aureus	14	9,7
Escherichia coli	36	25
Staphylocoques à coagulase négative (SCN)	9	6,2
Listeria monocytogenes	3	2,1
Autres entérobactéries	22	15,3

**Tableau 4:** Sensibilité de *Salmonella spp* aux antibiotiques (n= 60)

Antibiotiques	S (%)	I (%)	R (%)
Amikacine	46(76,6)	1(1,7)	13(21,7)
Amoxicilline	7(11,7)	-	53(88,3)
Augmentin	3(5)	-	57(95)
Ceftazidime	49(81,7)	-	10(18,3)
Ceftriaxone	48(80)	2(3,3)	10(16,7)
Cefuroxime	44(73,3)	-	16(26,7)
Chloramphénicol	5(8,3)	-	55(91,7)
Ciprofloxacine	55(91,7)	-	5(8,3)
Cotrimoxazole	6(10)	-	54(90)
Doxycycline	12(20)	4(6,7)	44(73,3)
Gentamicine	7(11,7)	-	53(32)
Négram	1(1,7)	-	59(98,3)
Norfloxacine	48(80)	-	12(20)

## Discussion

**Données sociodémographiques:** Les résultats de l'analyse descriptive du Tableau 1 montrent une prédominance des sujets dont l'âge est compris entre 16 et 30 ans avec une moyenne d'âge de 28,09 ± 17,72 ans; l'âge extrême était de 3 mois et 80 ans. L'analyse statistique montre une association entre le jeune âge (0-15 ans) et la FT. Le sexe masculin est prédominant avec un sexe ratio de 1,3. L'analyse montre aussi une association de la FT avec le sexe féminin (2 fois plus exposées que les hommes). Le Centre Hospitalier 8^ème^CEPAC Cahi a fourni plus des cas bien qu'aucune différence significative n'ait été notée entre les différents centres de prélèvement prouvant ainsi une distribution uniforme de la fièvre typhoïde tant au centre que dans la banlieue de la ville de Bukavu. Les sujets avec un niveau d'étude secondaire étaient plus nombreux dans l'étude. Cependant la FT est associée à un niveau d'étude bas. Nos résultats sont en partie en accord avec l'étude de Lefebvre et collaborateurs à Dakar, au Sénégal, qui en 2005 avaient rapporté un âge moyen des patients avec FT de 16,7±11,1 ans avec une médiane de 15 ans (extrêmes 1-52 ans). Le sexe ratio H/F était de 2,1 chez les enfants et de 0,9 chez les adultes [9]. Par contre au Liban une étude a montré que la répartition des cas de FT n'était pas homogène selon les différentes catégories d'âge; la tranche d'âge la plus touchée se situait entre 20-39 ans, suivie par la tranche de 10-19 ans [10]. Nos résultats rencontrent également en partie ceux de Lunguya et ses collaborateurs à Kinshasa qui avait trouvé que la tranche d'âge la plus touchée par la fièvre typhoïde était compris entre 10 et 19 ans en RDC [11]. Les résultats de notre deuxième tableau montrent que les signes cliniques associés à la fièvre typhoïde sont la fièvre, les vomissements et les douleurs abdominales. Ces résultats sont proches de ceux de Lefebvre et al. à Dakar, qui ont noté aussi la fièvre comme signe dominant les symptômes de la FT [9], de ceux de Lunguya et al. à Kinshasa, qui ont noté la fièvre et les douleurs abdominales [11], ainsi que de ceux de Gbadoé et al., qui au Togo ont trouvé une association entre FT et douleurs abdominales ou diarrhée [12].

**Sensibilité des *Salmonella spp* aux antibiotiques:** Il ressort de notre étude que les *Salmonelles* isolées chez nos patients avaient une bonne sensibilité aux quinolones (ciprofloxacine et norfloxacine), aux céphalosporines (céftazidime, cefuroxime et ceftriaxone) et à l'amikacine. Nous avons noté dans notre étude une diminution de la sensibilité au chloramphénicol, au cotrimoxazole, à l'amoxicilline, au Negram, à la doxicycline et à la gentamicine (Tableau 4). Ces résultats se rapprochent de ceux trouvés par par Lefebvre et al. à Dakar [9]. Cette diminution de la sensibilité aux antibiotiques précités a été trouvée en France par Weill [13]. Contrairement à l'émergence des souches résistantes à la Ciprofloxacine et aux céphalosporines de troisième génération trouvées en Asie par plusieurs auteurs [14, 15] le *Salmonelle spp* identifié à Bukavu garde encore une bonne sensibilité à la ciprofloxacine et aux céphalosporines de troisième génération.

## Conclusion

Les souches de *Salmonella spp* restent sensibles à la ciprofloxacine, au ceftazidime, à la ceftriaxone, à la norfloxacine, à l’amikacine et au cefuroxime avec un taux non négligeable de résistance. Ainsi, un test d'antibiogramme est requis pour une meilleure prise en charge de la fièvre typhoïde dans la ville de Bukavu.

### Etat des connaissances actuelle sur le sujet

- La fièvre typhoïde est le plus souvent rencontrée chez les jeunes dont l' âge est compris entre 10 et 19 ans;
- La fièvre et les douleurs abdominales sont les signes cliniques les plus f équemment rencontrés en cas de fièvre typhoïde;
- Il existe des souches de Salmonelles résistantes aux antibiotiques et même à la Ciprofloxacine.

### Contribution de notre étude à la connaissance

- Notre étude montre qu'en plus de la fièvre et les douleurs abdominales, à Bukavu la fièvre typhoïde est aussi associée à l'asthénie et au vomissement;
- Notre étude montre que les salmonelles sont responsables de plus de 60% des h émocultures positives;
- Notre étude montre queantibiotiques à utiliser à Bukavu en cas de fièvre typhoïde sont la ciprofloxacine, la ceftazidime et l'amikacine; les antibiotiques à éviter sont l'Augmentin, le chloramphénicol, le Negram, l'amoxycilline et le cotrimoxazole.

## Remerciements

Nous remercions beaucoup le Comité de Gestion de l'Université Evangélique en Afrique qui a soutenu financièrement cette étude. Nous remercions également tous les Médecins Directeurs, les Médecins, les Techniciens de Laboratoire et les malades des différentes formations sanitaires qui ont accepté de participer à cette étude en nous fournissant les échantillons et les données sociodémographiques et sanitaires.
